# Halophilic rhizobacteria promote growth, physiology and salinity tolerance in *Sesamum indicum* L. grown under salt stress

**DOI:** 10.3389/fmicb.2025.1590854

**Published:** 2025-05-14

**Authors:** Dharman Sridhar, Saleh S. Alheswairini, Jayanthi Barasarathi, Hesham Ali El Enshasy, Sundaram Lalitha, Sajad Hussain Mir, S. Nithyapriya, Riyaz Sayyed

**Affiliations:** ^1^Department of Botany, School of Life Sciences, Periyar University, Salem, India; ^2^Department of Plant Protection, College of Agriculture and Food, Qassim University, Buraidah, Saudi Arabia; ^3^Faculty of Health and Life Sciences (FHLS), Inti International University, Nilai, Malaysia; ^4^Innovation Centre in Agritechnology for Advanced Bioprocessing (ICA), Universiti Teknologi Malaysia, Johor Bahru, Malaysia; ^5^Faculty of Chemical and Energy Engineering, Universiti Teknologi Malaysia (UTM), Johor Bahru, Malaysia; ^6^PG and Research Department of Botany, Padmavani Arts and Science College for Women, Salem, India; ^7^Key Laboratory of Integrated Crop Pest Management of Anhui Province, College of Plant Protection, Anhui Agricultural University, Hefei, Anhui, China; ^8^Department of Biological Sciences and Chemistry, University of Nizwa, Nizwa, Oman

**Keywords:** PGPR traits, *B. flexus*, metabolites, antioxidant, salt stress

## Abstract

**Introduction:**

Salt stress is a major global issue that negatively affects plant growth and physiological processes. Plant growth-promoting rhizobacteria (PGPR) are known to alleviate salt stress and promote plant growth. This study aimed to isolate and characterize salt-tolerant PGPR from salinity-affected soils in Tamil Nadu, India, and assess their potential to enhance growth and salt tolerance in sesame (*Sesamum indicum* L.).

**Methods:**

Salt-tolerant PGPR were isolated and screened for plant growth-promoting traits. One isolate, designated PAS1, demonstrated significant capabilities, including the production of indole-3-acetic acid (IAA; 48.56 μg ml^−1^), siderophore production (89.20 ± 0.65%), phosphate solubilization (7.8 mm zone of clearance), ammonia, and hydrogen cyanide (HCN) production. PAS1 was identified as *Bacillus flexus*. Sesame plants were inoculated with *B. flexus* and grown under different salt concentrations (0, 100, and 200 mM NaCl) for 45 days.

**Results:**

Inoculation with *B. flexus* significantly improved the biochemical parameters of sesame plants under salt stress, including increased chlorophyll content (4.4 mg g^−1^), proline (0.0017 mg g^−1^), soluble sugars (61.34 mg g^−1^), amino acids (1.10 mg g^−1^), and proteins (3.31 mg g^−1^). Additionally, antioxidant enzyme activities were enhanced, as indicated by DPPH scavenging activity (60.25%), superoxide dismutase (231.29 U mg g^−1^ protein), peroxidase (6.21 U mg g^−1^ protein), catalase (3.38 U mg g^−1^ protein), and a reduction in malondialdehyde (23.32 μmol g^−1^).

**Discussion:**

The study demonstrates that inoculation with salt-tolerant *B. flexus* can effectively improve sesame plant growth and enhance tolerance to salt stress. These findings suggest that halo-tolerant PGPR strains like *B. flexus* could serve as promising biofertilizers to improve crop productivity in salt-affected agricultural soils.

## Graphical Abstract

Role of *Bacillus flexus* in enhancing sesame growth under salt stress through PGPR mechanisms and improved physiological responses. 
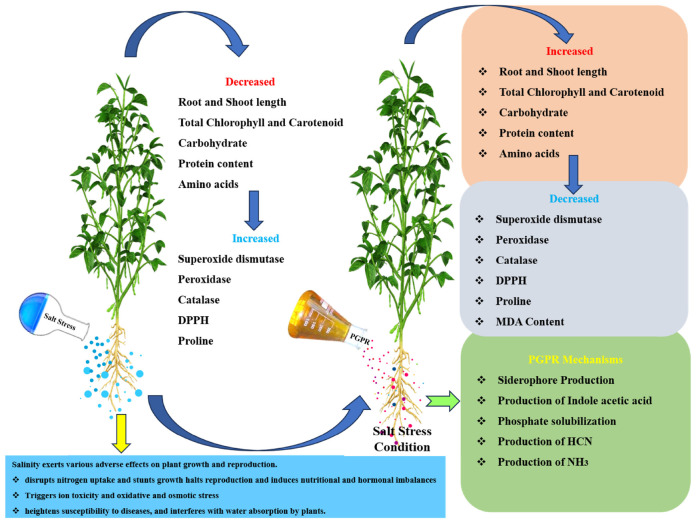

## Highlights

• Salt-tolerant PGPR strain protects inoculated sesame plants against salinity stress.
• The effect of PAS1 treatment on sesame plant showed restoration of physiological traits from salinity stress condition to normal condition.
• *Bacillus flexus* inoculation increased antioxidant defense mechanisms under salt conditions.
• The findings of this study strongly support future efforts to manage plant salinity stress with the development of effective bioinoculants for agricultural use.

## Introduction

Climate change is the most pressing challenge of our era, impacting the earth in diverse ways. Rising sea levels, driven by climate change, are causing saltwater intrusion into agricultural lands, increasing soil salinity. This salinization ultimately contributes to global food insecurity and diminished agricultural productivity (Khan et al., 2021; Mukhopadhyay et al., 2021; Gulzar et al., 2025). Globally, 20% of irrigated lands are severely damaged by salinity (Singh, 2022; Patel et al., 2023), and this land degradation is projected to reach 50% by 2050 (Dias et al., 2021). Approximately 70% of yield loss in cereal crops, including wheat, rice, sorghum, sesame, and barley, is attributed to soil contamination from salinity and sodicity (Sagar et al., 2022a; Awaad, 2023).

Salinity, an abiotic chemical stress, is characterized by the accumulation of soluble salts in the rhizosphere, adversely affecting plant metabolism in two primary ways (Kalam et al., 2020; Khan et al., 2021). First, high salt concentrations create hyper-osmotic and hypertonic environments that damage the root system, impairing water and nutrient uptake (Seleiman et al., 2022; Zhang et al., 2024). This leads to secondary stresses, such as oxidative stress, which induces membrane instability through DNA and protein denaturation and lipid peroxidation. Ultimately, these effects trigger programmed cell death (apoptosis) and cause the deterioration of the entire plant (Debnath et al., 2021; Paes de Melo et al., 2022).

Remediation of saline soils traditionally involves inorganic amendments (e.g., gypsum, lime, sulfuric acid derivatives, and synthetic fertilizers) and organic amendments (e.g., green manure, farmyard manure, and industrial wastes such as press mud) (Kapadia et al., 2021; Kour et al., 2021). However, these methods have shown limited success in improving stress tolerance in economically important crops under field conditions due to several factors (Soto-Gómez and Pérez-Rodríguez, 2022). Plant growth-promoting rhizobacteria (PGPR) has recently gained attention as a sustainable and eco-friendly approach to enhance crop production in salt-affected lands (Nasab and Sayyed, 2019; Sagar et al., 2022b; Kapadia et al., 2022; Jabborova et al., 2025). These beneficial bacteria colonize plant roots, thrive in hyper-saline environments, and improve plant growth and yield (Choudhary et al., 2022; Kumawat et al., 2022). Rhizobacteria have been reported to promote plant growth effectively, even under saline conditions (Ayuso-Calles et al., 2021; Basu et al., 2021). Halophilic and halo-tolerant PGPR employ several mechanisms to withstand severe environmental stresses and minimize yield loss (Hernández-Canseco et al., 2022). These include synthesizing osmolytes to maintain cellular osmotic balance, regulating ion transporters to reduce the toxic effects of Na^+^ and Cl^–^ ions, and activating plant defense systems to scavenge reactive oxygen species (ROS) and alleviate oxidative stress (Kusale et al., 2021; Chattha et al., 2022). Furthermore, these microorganisms produce phytohormones, fix nitrogen, solubilize phosphate, generate volatile compounds, and produce antifungal or antibacterial metabolites (Hamid et al., 2021; Koza et al., 2022; Dave et al., 2024; Praveen et al., 2024; Anbalagan et al., 2025; Bright et al., 2025). They also aid in mobilizing mineral ions, enhancing photosynthesis, and regulating osmotic pressure through the heterodimerization of acids (Burnap, 2023; Sethi et al., 2025). Additionally, PGPR contribute to plant defense by producing antioxidants, betaines, and other compounds that mitigate the harmful effects of ROS and toxins (Sagar et al., 2020; Hasanuzzaman et al., 2021).

*Sesamum indicum* L. is a valuable oilseed crop cultivated widely in India, China, Thailand, Mexico, Guatemala, El Salvador, Afghanistan, Pakistan, Bangladesh, Indonesia, Sri Lanka, Saudi Arabia, and Turkey (Morris et al., 2023). Sesame seeds are highly valued for their rich oil, protein, and antioxidant content, making them essential in food, nutraceuticals, pharmaceuticals, and various industries (Wei et al., 2022). They offer health benefits such as reducing cancer risk, mitigating mucosal, colon, and liver damage, lowering serum cholesterol levels, and improving vitamin E activity and α-tocopherol availability (Zainal et al., 2022). Sesame seeds also help reduce thiobarbituric acid reactive substances (TBARS) (Mitsiopoulou et al., 2021). Lignan in sesame seeds acts as a phytoestrogens and is converted to enterolactone, which is crucial in preventing hormone-dependent cancers (e.g., breast and prostate) and cardiovascular diseases (Jang et al., 2022).

The present study aimed to evaluate the role of halo-tolerant plant growth-promoting (PGP) microorganisms in sesame. Plant parameters for evaluation include the enhancement of growth and yield and morpho-physiological and biochemical traits. The findings will help explore the potential of salt-tolerant PGPR strains as biofertilizers to mitigate yield loss caused by salt stress in sesame crops. Ultimately, this research may contribute to developing effective biomass solutions for managing complex, saline soils.

## Materials and methods

### Isolation of potent PGPR

Plant growth promoting rhizobacteria cultures were isolated from the saline soil of Kanniyakumari, Tamil Nadu, India (8°09′′20′′N, 77°27′27′′E). Using the serial dilution method, 100 μL aliquots were spread onto nutrient agar (NA) plates, the plates were incubated at 30 ± 2°C for 24 h. Morphologically distinct bacterial colonies were selected, isolated, and purified. The purified isolate designated PAS1 was used for further studies.

### Gram-staining and biochemical properties of the isolate

Purified culture of each isolate was stained using Gram’s staining and observed under an oil immersion microscope at 100 × magnification (Coico, 2006). The catalase activity of the bacterial isolates was tested according to Whittenbury (1964) method. A small amount of bacterial colony was mixed in a drop of 3% hydrogen peroxide and observed for the evolution of gas bubbles as an indication of a positive catalase test. The ability of the isolate to utilize citrate as the sole carbon source was assessed using Simmons citrate agar medium, as described by MacWilliams (2009). The inoculated plates were incubated at 30°C for 7 days and observed for color change from green to blue as an indication of a positive citrate test.

### Evaluation of plant growth-promoting traits

#### Siderophore production

The Chrome Azurol Sulfonate (CAS) agar plate method was used to assess siderophore production (Alexander and Zuberer, 1991; Patel et al., 2018). Bacterial isolates were grown on CAS at 30°C for 24–48 h and observed for the development of an orange halo around the colonies. For quantitative estimation, bacterial culture was grown in succinate medium at 30°C for 24–48 h, and the amount of siderophore was estimated according to Himpsl and Mobley (2019) and expressed as siderophore units (SU), using the following formula.


$$\%\mathrm{SU}=\frac{\mathrm{As}-\mathrm{Ar}}{\text{Ar}}$$

where As is the absorbance of the sample (CAS reagent mixed with the bacterial supernatant), and Ar is the absorbance of the reference (CAS reagent mixed with uninoculated medium).

### Indole acetic acid production

Production of IAA by isolates was determined using the method of Gordon and Weber (1951). The bacterial isolates were grown in Luria and Bertani (LB) broth enriched with tryptophan (100 μg ml^–1^) at 30 ± 2°C for 72 h. These culture supernatants from the isolate were combined with Salkowski’s reagent in a 1:2 ratio and observed for the development of a pink color as an indication of IAA production. The amount of IAA produced was measured spectrophotometrically at 530 nm, using an IAA calibration curve (10–100 μg ml^–1^).

### Ammonia production

For this, 24 h old bacterial cultures were individually grown in 10 ml of peptone at 30 ± 2°C for 48 h. Following incubation, 0.5 ml of Nessler’s reagent was added, and observed for color change from brown to yellow as a signal of the presence of ammonia (Hills, 1940).

### Hydrogen cyanide production

Hydrogen cyanide production was assessed using the procedure described by Lorck (1948). Bacterial isolates were inoculated into LB broth containing glycine, and Whatman No. 1 filter paper soaked in a solution of 0.5% C_6_H_3_N_3_O_7_ and 2% Na_2_CO_3_ was placed on top of the inside of the tubes. A color change of the filter paper from orange to red indicated HCN production.

### Phosphate solubilization

Phosphate (P) solubilization was determined using Pikovskaya’s agar medium (Nautiyal, 1999). Bacterial colonies were spot-inoculated at the center of the plates and incubated for 7 days at 30°C and observed for the formation of clear halo zones of P solubilization around the colonies. The P solubilization ability was quantified by calculating the Solubilization Index (SI) using the following formula:


$$\text{SI}=\frac{\mathrm{Colony}\,\mathrm{diameter}+\mathrm{Halo}\,\mathrm{zone}\,\mathrm{diameter}}{\mathrm{Colony}\,\mathrm{diameter}}$$

### Identification of isolate through 16s rRNA sequencing

Genomic DNA was extracted from the screened multifunctional PGPB strain using a commercial bacterial genomic DNA extraction kit, following the manufacturer’s instructions. The extracted DNA was used as a template for polymerase chain reaction (PCR) amplification of the 16s rRNA gene, using universal bacterial primers 27F (5′-AGAGTTTGATCCTGGCTCAG-3′) and 1492R (5′-GGTTACCTTGTTACGACTT-3′). The PCR reaction mixture (25 μL) contained 1 μL of DNA template, 0.5 μL of primer 27F, 0.5 μL of primer 1492R, 12.5 μL of 2x Taq PCR Master Mix, and 10.5 μL of nuclease-free water. PCR conditions were as follows: initial denaturation at 93°C for 3 min, followed by 32 cycles of denaturation at 93°C for 30 s, annealing at 56°C for 30 s, and extension at 72°C for 2 min with a final extension at 72°C for 7 min. The amplified PCR products were subjected to bidirectional sequencing by BGI. The obtained sequences were assembled and analyzed using BLAST against the NCBI GenBank database for species-level identification. A phylogenetic tree was constructed using the neighbor-joining method in MEGA version 11.0, and the reliability of the tree was assessed through bootstrap analysis with 1,000 replicates (Sanschagrin and Yergeau, 2014).

### Pot experiment design

Various physicochemical properties of the soil were assessed according to the methods described by Jackson (1985). The selected bacterial isolate *B. flexus* (PAS1) was grown in LB broth at 28 ± 1°C for 24 h under a shaking at 100 rpm. After incubation, the bacterial culture was applied to the soil in pots.

Treatment 1: Control

Treatment 2: Control+PAS1

Treatment 3: NaCl 100 mM

Treatment 4: NaCl 100 mM+PAS1

Treatment 5: NaCl 200 mM

Treatment 6: NaCl 200 mM+PAS1

The experiment included treatments with *B. flexus* (PAS1) in control soil and soils supplemented with NaCl at concentrations of 0 mM, 100 mM, and 200 mM, with each treatment replicated three times. Sesame seeds (TMV7) were soaked in the PAS1 inoculum for 1 h before being sown in the treated pots. The physicochemical properties of the test soil were pH 8.02, EC 3.17 dS/m, K+ 62 kg/acre, Na+ 10.12 me/L, SAR 5.72 mmolc/L, and ESP 8.12%. Based on these properties, the soil was classified as saline. Plants were harvested after 45 days, and root and shoot length measurements, metabolites, and antioxidant enzymes were recorded.

### Seed dormancy, germination, and morphological traits

Sesame seeds were surface sterilized and plated on 0.6% agar (pH 5.7) for 1 h. The seeds were then exposed to light conditions for 4 days, and germination frequency was recorded. For light-mediated germination assays, seeds were stored dry at 25°C for 2–5 months before being surface sterilized, plated on 0.6% agar with 0.01% (v/v) ethanol (mock) for 1 h, and treated with salt stress (Bentsink and Koornneef, 2008). The growth of the radical defines germination. After 45 days, the growth of sesame seedlings was examined, and growth parameters such as root length and shoot length were measured.

### Determination of chlorophyll content and carotenoids

The total chlorophyll content and carotenoids in the fresh leaves of plants were determined using the method outlined by Arnon (1949).


Total⁢chlorophyll⁢(mg/g)= 20.2⁢(A⁢645)- 8.02⁢(A⁢663)

Carotenoids⁢(mg/g)=5.02⁢(A⁢480)

### Primary metabolites

#### Estimation of carbohydrate

The total carbohydrate content of the leaf was determined using the protocol Hedge and Hofreiter (1962). 0.5 g of dried leaves were ground with 10 mL of 1 N sulfuric acid, then transferred to a test tube and heated at 100°C for 24 h. Then, 1 mL of sugar solution was mixed with 1 mL of 5% phenol solution, after which 5.0 mL of sulfuric acid was added. Incubation was carried out for 20 min at 23–30°C. The total carbohydrate content was measured at 490 nm.

#### Estimation of protein

The total protein content in the leaves was measured following the method of Bradford (1976). Plant leaves were first powdered in liquid nitrogen and then homogenized with a 50 mM Na-phosphate buffer (pH 7.6). The homogenate was centrifuged at 6,000 × *g* for 20 min at 4°C, and the absorbance of the supernatant was recorded at 595 nm. The protein content was estimated and the standard curve was created using the Bovine Serum Albumin (BSA) standards.

### Estimation of amino acids

The leaf extract was blended with 3 mL of 80% (v/v) methanol and subjected to incubation in hot water at 70°C for 30 min. Following this, an equal volume of 5% phenol and 1.5 ml of concentrated H_2_SO_4_ was introduced to the mixture, which was subsequently incubated in the dark for an additional 30 min. The absorbance of the resulting reaction mixture was measured at 490 nm (Roland and Gross, 1954).

### Estimation of proline

The proline content in fresh leaves was determined as per Bates (1973). Fresh leaves (0.1 g) were homogenized in 3 mL of 3% sulfosalicylic acid and heated at 95°C for 15 min. The homogenate was then centrifuged at 8,000 × *g* for 10 min. The supernatant was mixed with equal glacial acetic acid, 2% acid ninhydrin, and 6 M orthophosphoric acid. This mixture was heated in a boiling water bath for 30 min and then cooled to room temperature for 30 min. After adding 1 mL of toluene and shaking vigorously for 30 s, the absorbance of the upper toluene phase was measured at 520 nm using toluene as a blank. Free proline content was determined by comparing the absorbance to a standard curve of pure L-proline and was calculated based on fresh weight, expressed as mg per 100 g FW.

### Antioxidant enzyme activities

#### Scavenging of the DPPH radical analysis

2,2-diphenyl-1-picrylhydrazyl (DPPH) radical scavenging activity was assessed according to the method described by Brand-Williams et al. (1995). A total of 1 g of plant powder was mixed with 2 mL of 80% (v/v) methanol and shaken for 24 h at 25°C. The mixture was then centrifuged at 12,000 × *g* for 20 min, and the supernatant was collected. This supernatant was combined with an equal volume of DPPH solution (0.1 M), and the reaction mixtures were incubated in the dark for 40 min. The absorbance was then measured at 515 nm.

### SOD, CAT, and POD enzyme activities

Superoxide dismutase (SOD) activity was assessed following the method outlined by Flohé and Ötting (1984). One unit of the enzyme is defined as the amount of SOD that inhibits 50% of nitro blue tetrazolium at 25°C. The reaction mixture consisted of 50 mM sodium phosphate buffer (pH 7.6), 13 mM methionine, 75 μM NBT, 2 μM riboflavin, 0.1 mM EDTA, and 0–50 μL of enzyme extract, with riboflavin being added last. The tube was then shaken and exposed to a 40-watt fluorescent lamp for 15 min, and absorbance was subsequently measured at 560 nm.

Catalase (CAT) activity was measured by converting H_2_O_2_ to water and oxygen, following the method described by Lück (1965a). For the peroxidase (POD) activity assay, the reaction mixture consisted of 750 μL of 50 mM phosphate buffer, 100 μL of 20 mM guaiacol, 100 μL of 40 mM H_2_O_2_, and 100 μL of enzyme extract. The absorbance of the mixture was recorded every 20 s at 470 nm for 3 min (Lück, 1965b).

### Estimation of lipid peroxidation

Two grams of fresh leaf tissue was homogenized in 600 μL of 0.1% trichloroacetic acid (TCA). The homogenate was centrifuged at 15,000 × *g* for 20 min at 4°C. A total of 1.5 mL of 20% TCA containing 0.5% Thio barbituric acid (TBA) was added to the supernatant, and the mixture was heated at 95°C for 25 min. After heating, the mixture was cooled and centrifuged at 15,000 × *g* for 5 min at 4°C. The absorbance of the supernatant was then measured at 532 nm. The results were expressed as μmol MDA per gram of fresh weight (FW) (Moore and Roberts, 1998).

### Data analysis

The experiments were conducted in triplicate (*n* = 3). Graphs were generated using Prism software version 5.01 (Graph Pad Software Inc., United States), and data were analyzed using SPSS 16.0 employing One-way ANOVA. Statistical significance was determined using Tukey’s multiple comparison tests (*P* < 0.05).

## Results

### Isolation of salt-tolerant PGPR strains and identification of PGPR characteristics

Bacterial isolate obtained from high-salinity areas of the Kanyakumari district, India, produced siderophore production, IAA, ammonia, HCN, and solubilized phosphate. It was Gram-positive, motile, rod and exhibited catalase activity and utilized citrate (Supplementary Table 1).

### Analysis of 16s rRNA sequencing followed by a phylogenetic tree

**FIGURE 1:**
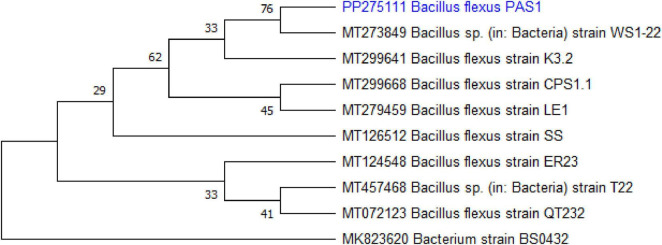
Phylogenetic tree based on 16s rRNA gene sequences of *Bacillus flexus*.

PCR amplification of the 16s rRNA gene was carried out for the identification of the selected bacterial isolate. The amplified sequence was analyzed through the NCBI BLAST search and has been submitted to the GenBank (NCBI accession number: PP275111). The isolate was identified as *Bacillus flexus*. A phylogenetic tree was constructed based on the 16s rRNA gene sequence using the neighbor-joining method in MEGA version 11.0. The tree indicated that the isolate belongs to the genus *Bacillus* and clustered closely with *B. flexus*, thereby confirming its identity (Figure 1).

### Effect of *B. flexus* on salt stress on *S. indicum* growth parameters

**FIGURE 2:**
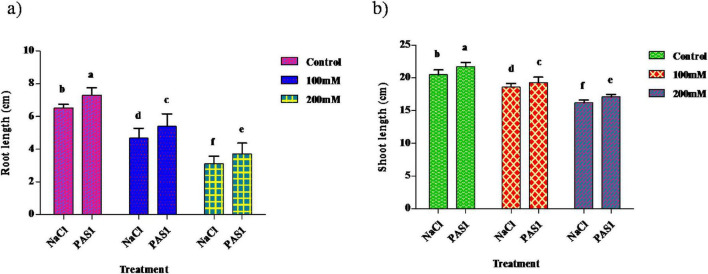
The effect of plant growth-promoting rhizobacteria (PGPR) *B. flexus* (PAS1) on plant growth parameters was evaluated in pots subjected to salt stress. The parameters measured were root **(a)** and shoot **(b)** length. Error bars represent the standard deviation (*n* = 3). Small alphabetical letters above the error bars indicate significant differences between treatments according to Tukey’s *post-hoc* test (*p* = 0.05).

The plant was grown in different concentrations of NaCl (Control, 100 mM, and 200 mM). The shoot lengths of the inoculated were 21.7, 19.3, and 17.1, and the shoot lengths of the non-inoculated were 20.5, 18.6, and 16.2. This inoculated strain gave good results when grown under NaCl compared to the control (Figure 2a). Similar to shoot length, the root length of the inoculated was 7.3, 5.4, 3.9, and the root length of the non-inoculated was 6.5, 4.7, and 3.1. As mentioned above, root length in inoculation showed better results than the control (Figure 2b).

### Determination of total chlorophyll (TC) and carotenoids in *S. indicum* with *B. flexus* under salt stress

**FIGURE 3:**
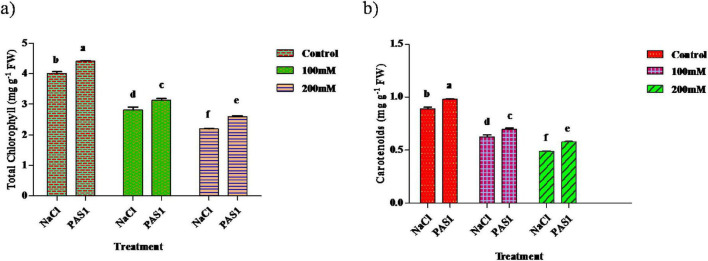
The effect of plant growth-promoting rhizobacteria (PGPR) *B. flexus* (PAS1) on photosynthetic pigments, total chlorophyll **(a)**, and carotenoid **(b)** levels under NaCl stress in sesame leaves was investigated. Error bars represent the standard deviation (*n* = 3). Small alphabetical letters above the error bars indicate significant differences between treatments according to Tukey’s *post-hoc* test (*p* = 0.05).

Chlorophyll is essential in photosynthesis and is plants’ light energy source. As soil salinity increases, chlorophyll decreases. The chlorophyll content in the plant was higher in the inoculated samples (4.4, 3.1, and 2.6 mg g^–1^) compared to the control (4.0, 2.8, and 2.1 mg g^–1^) (Figure 3a). Similarly, a substantial increase in carotenoid content was (0.97, 0.69, and 0.57 mg g^–1^) was evident over the control (0.29, 0.62, and 0.48 mg g^–1^) (Figure 3b). However, the amount of chlorophyll pigments and carotenoid content decreased with increasing salinity.

### Determination of primary metabolites in *S. indicum* with *B. flexus* under salt stress

**FIGURE 4:**
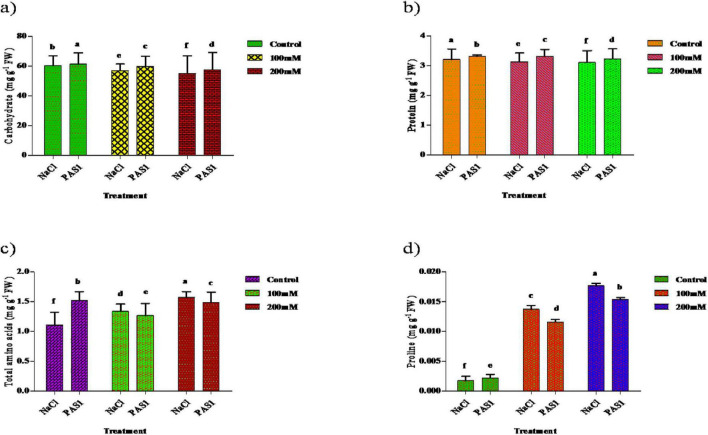
The effect of plant growth-promoting rhizobacteria (PGPR) *B. flexus* (PAS1) on total carbohydrate **(a)**, protein **(b)**, amino acids **(c)**, and proline **(d)** levels under NaCl stress in sesame leaves was investigated. Error bars represent the standard deviation (*n* = 3). Small alphabetical letters above the error bars indicate significant differences between treatments according to Tukey’s *post-hoc* test (*p* = 0.05).

Regarding carbohydrate content, a significant increase was observed in inoculated plants compared to control. These are 61.34, 59.83, 57.53,60.27, 57.06, and 55.03 mg g^–1^ inoculated and non-inoculated, respectively (Figure 4a). Also, the protein level was higher in inoculated plants (3.31, 3.22, and 3.10 mg g^–1^), than in untreated (control) plants 3.20, 3.13, and 3.08 mg g^–1^ (Figure 4b). Amino acid concentration also increased in plants grown under salt stress. A higher amino acid content was recorded in PGPR treated plants (1.15, 1.33, and 1.57 mg g^–1^) than control plants (1.15, 1.26, and 1.48 mg g^–1^) (Figure 4c). Higher levels of proline contents were observed in PGPR treated plant grown under salinity to be 0.0176, 0.0137, 0.0017 mg g^–1^ higher in untreated plants. Also, treated plants exhibited lower proline levels with values of 0.0153, 0.0115, and 0.0022 mg g^–1^ compared to the control (Figure 4d).

### Determination of antioxidant enzyme activity in *S. indicum* with *B. flexus* under salt stress

**FIGURE 5:**
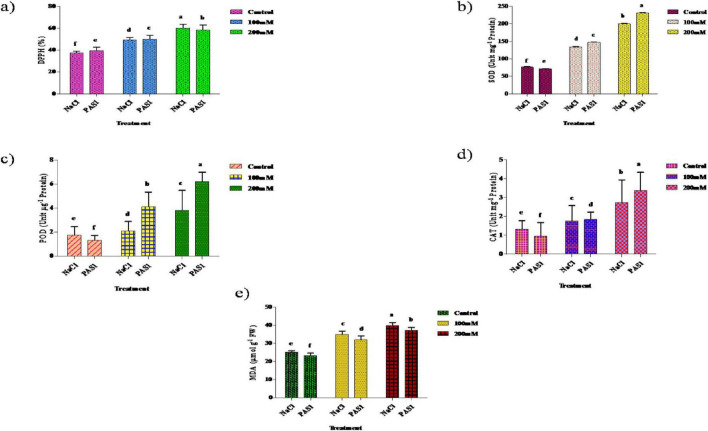
The effect of plant growth-promoting rhizobacteria (PGPR) *B. flexus* (PAS1) on DPPH % **(a)**, SOD **(b)**, POD **(c)**, CAT **(d)**, and MDA **(e)** content levels under NaCl stress in sesame leaves was investigated. Error bars represent the standard deviation (*n* = 3). Small alphabetical letters above the error bars indicate significant differences between treatments according to Tukey’s *post-hoc* test (*p* = 0.05).

2,2-diphenyl-1-picrylhydrazyl activity increased significantly in both control and PGPR treatments. Specifically, the activity increased by 23.8% at 100 mM NaCl and 59.5% at 200 mM NaCl, while the PAS1-treated showed increases of 13.6% and 45.5%. SOD, POD, and CAT activities were also higher in NaCl-treated plants compared to control. Significant increases were observed for POD, SOD, and CAT with 5.44%, 13.64%, and 31.14% increases in NaCl treatment of 0, 100, and 200 mM, respectively. The lowest MDA levels were observed in control plants, with 25.6 μmol g-^1^ FW for NaCl and 24.1 μM g^–1^ FW for PGPR treatments. At 100 mM NaCl, MDA increased to 34.7 μmol g^–1^ FW in NaCl-treated plants and 32.5 1 μmol g^–1^ FW in PGPR-treated plants. At 200 mM NaCl, MDA levels increased to 42.3 μmol g^–1^ FW and 39.6 μmol g^–1^ FW, respectively. The increase in MDA was lower in PGPR-treated plants compared to untreated plants. Co-application of *B. flexus* with NaCl stress further enhanced the antioxidant enzyme activity observed with NaCl stress. The activity of antioxidant enzymes, including SOD, POD, CAT, and MDA content under NaCl stress, significantly increased during the experiment in cultivars compared to the control (Figure 5).

### Pearson’s correlation coefficient analysis

**FIGURE 6:**
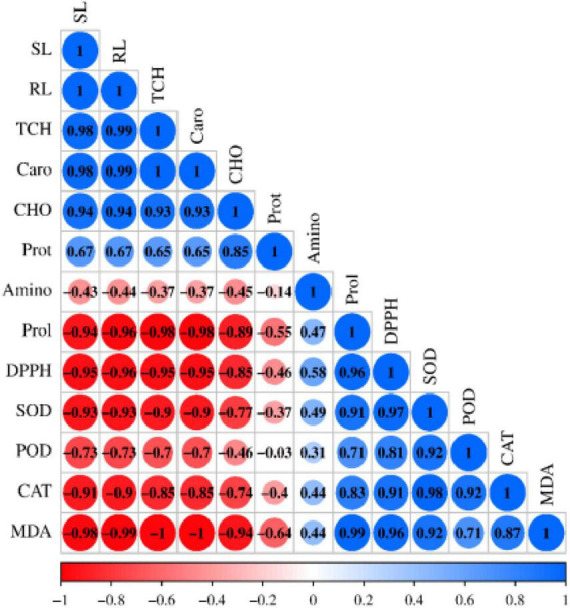
Pearson’s correlation analysis was performed on the morphological, physiological, and biochemical attributes of sesame treated with plant growth-promoting rhizobacteria (PGPR) under salt stress. The attributes analyzed include SL (shoot length), RL (root length), TCH (total chlorophyll), Caro (carotenoids), CHO (carbohydrate), Prot (protein), Amino (amino acid), Prol (proline), DPPH, SOD, POD, CAT, and MDA (lipid peroxidation).

A Pearson’s correlation analysis was performed to determine the relationship between morphological, physiological, biochemical, and antioxidant traits under salt stress (Figure 6). The analysis revealed a strong, significant positive correlation (*p* < 0.01) among shoot length (SL), root length (RL), total chlorophyll (TCH), carotenoids, carbohydrate content (CHO), and protein content. This indicates that these parameters are positively associated with each other and play a major role in plant growth promotion. Similarly, a significant positive correlation (*p* < 0.01) was observed among amino acid content, proline accumulation, DPPH activity, SOD, POD, CAT, and MDA. In contrast, a significant negative correlation (*p* < 0.05) was found between growth parameters (SL and RL) and stress such as proline, DPPH, SOD, CAT, and MDA. TCH and carotenoids also negatively correlated with proline, DPPH, SOD, and MDA, while CHO was negatively correlated with MDA. Additionally, negative correlations were noted between SL, RL and amino acids, POD; TCH and carotenoids with amino acids, POD, CAT; CHO with amino acids, proline, DPPH, SOD, POD, CAT; and protein with all stress including amino acids, proline, DPPH, SOD, POD, CAT, and MDA.

## Discussion

Various crop plants thrive in diverse regions worldwide, often overcoming challenges from varying climatic conditions, soil types, and pests. Among these challenges, salt stress is a significant abiotic stress that significantly impacts agriculture (Ashraf and Munns, 2022; Al-Turki et al., 2023). This study analyzed soil samples collected from the rhizosphere of plants growing in saline-affected soil. PGPR are soil-dwelling microorganisms that enhance plant growth through direct or indirect mechanisms during root colonization and contribute to increased crop yields (Rai et al., 2023; Saini et al., 2023). Several studies have identified beneficial PGPR based on their growth-promoting properties (Passari et al., 2016; Singh et al., 2019; Bright et al., 2025; Sethi et al., 2025). For example, various *Pseudomonas* and *Bacillus* strains promote plant growth by producing indole-3-acetic acid (IAA), stimulating root tip growth, and enhancing phototropism (Paravar et al., 2023; Sagar et al., 2024). PGPR can also mitigate salinity stress and improve nitrogen uptake by plants (Kapadia et al., 2021; Rana et al., 2022). Phosphate solubilization, mediated by the release of inorganic and organic acids, converts insoluble phosphate in the soil into orthophosphate, making it available for plant uptake (Parray et al., 2023; Yu et al., 2024). Siderophore, produced by PGPR, facilitates iron acquisition by plants through the solubilization of organic and inorganic minerals in the soil (Pattnaik et al., 2021; Omar et al., 2022), thereby promoting root and shoot elongation (Meena et al., 2020). Furthermore, PGPR can control soil-borne pathogens by producing hydrogen cyanide (HCN), protecting plants from diseases, particularly root damage, and indirectly promoting plant growth (Sehrawat et al., 2022). Our study demonstrated that the isolated PGPR produced IAA, solubilized phosphate, and generated ammonia, siderophores, and HCN, consistent with the findings of (Mazumdar et al., 2020). The bacterial isolate was identified as *B. flexus* based on 16s rRNA gene sequencing, and a phylogenetic tree was constructed using the neighbor-joining method (Kapadia et al., 2022; Zhou et al., 2022). Previous studies have reported significant reductions in root and shoot length in various crop plants under salt stress (Kumar et al., 2021; Vafa et al., 2024). PGPR induce physiological changes in plant tissues, promoting growth and improving various plant parameters (Chauhan et al., 2022; Bright et al., 2025). For example, a study on wheat treated with *P. fluorescens* and *B. licheniformis* demonstrated improved growth under saline conditions (Orozco-Mosqueda et al., 2020). Similarly, *Azospirillum lipoferum*, *Azospirillum brasilense*, and *Bacillus* spp. have been shown to enhance plant growth under different salt stress concentrations (0, 50 mM) (Rabiei et al., 2020; Sagar et al., 2022b). Our results are consistent with those of Jalil and Ansari (2020), who demonstrated that plants protect themselves against salt stress by producing compatible solutes such as carbohydrates, proteins, and amino acids. These solutes are responsible for intracellular osmotic adjustment, production of antioxidant enzymes, excess ROS reduction, and membrane stability maintenance (Parveen et al., 2021; Hamidian et al., 2023). Plants employ various conservation strategies to enhance growth and counteract the detrimental effects of salt stress (Nasab and Sayyed, 2019; Ayub et al., 2020). In our study, proline concentration was higher under salt stress, similar to the findings of Patriarca et al. (2021). Proline has been shown to reduce enzymatic degradation induced by NaCl and other stresses in plants, thereby reducing the activity of antioxidant enzymes (Shahid et al., 2022). Proline acts as an osmoprotectants and a scavenger of hydroxyl radicals (Hosseinifard et al., 2022), ensuring membrane and sub-cellular structural integrity (Maslennikova et al., 2023). It also protects cellular functions by scavenging ROS (Feng et al., 2024). The DPPH scavenging activity of plant extracts indicated the presence of antioxidant activity. In our study, both plant and leaf extracts exhibited DPPH scavenging activity under salt stress, similar to the observations of Azeem et al. (2023). One of the primary plant defense strategies against reactive oxygen species (ROS) involves buffering ROS levels. Plants employ a coordinated defense mechanism involving antioxidant enzymes such as SOD, POD, and CAT (Kesawat et al., 2023). The activities of CAT, SOD, and POD in salt-stressed sage plants in our study were consistent with those reported by Hussain et al. (2023). Furthermore, the application of NaCl enhanced the activities of CAT, SOD, and POD, indicating oxidative damage in salt-stressed sage, similar to observations in other crops (Sheikhalipour et al., 2024). Lipid peroxidation, indicated by MDA levels, signifies membrane damage and leakage under salt stress (Syeed et al., 2021). Consistent with our findings, low MDA levels have been associated with salt tolerance in various studies. For example, salt-tolerant tomato cultivars (Khan et al., 2019; Raja et al., 2022) and salt-resistant tobacco plants (Wang et al., 2022) exhibited reduced lipid peroxidation, reflecting their ability to minimize oxidative damage under salinity. Salt stress can induce the biosynthesis of endogenous nitric oxide (NO), which acts as a direct ROS scavenger or a signaling molecule, thus reducing ROS levels and oxidative damage in stressed plants (Hasanuzzaman et al., 2021; Gao et al., 2022). PGPR improves the characteristics of crop plants and oilseeds and enhances yield (Kusale et al., 2021; Nagrale et al., 2023). Each PGPR strain uniquely supports plant growth in various polluted environments, contributing significantly to plant growth and product quality. The findings of this study strongly suggest that rhizobacteria, which produce PGPR traits and possess plant growth-promoting properties, play a key role in enhancing the ability of oil-yielding plants like sesame to adapt to saline environments.

## Conclusion

The study revealed that *B. flexus* from the contaminated area was resistant to NaCl. It enhances sesame growth under salt stress, which improves photosynthesis, transpiration, and photosynthetic pigments. In addition, *B. flexus* facilitates the availability of essential nutrients such as nitrogen, calcium, and iron in the soil. *B. flexus* halo-tolerant PGPR strains act as biofertilizers when cultivated in saline soils under salt-stressed conditions. This highlights the potential of improving these PGPR strains to mitigate the adverse effects of salt stress on plant growth and promote sustainable agriculture in salt-affected areas. In conclusion, this study suggests the critical role of *B. flexus* as a halo-tolerant PGPR in mitigating salt stress and promoting plant growth. These findings have considerable potential for practical applications in agricultural systems such as sustainable agriculture.

## Data Availability

The original contributions presented in this study are included in this article/Supplementary material, further inquiries can be directed to the corresponding authors.
