# Supplementary material for: Halophilic rhizobacteria promote growth, physiology and salinity tolerance in Sesamum indicum L. grown under salt stress
Source: Front Microbiol. 2025 May 14;16:1590854. doi: 10.3389/fmicb.2025.1590854 (PMC12116546; doi:10.3389/fmicb.2025.1590854)
Supplement: Supplementary file 1 [file Data_Sheet_1.docx]

**Supplementary Table 1.** PGPR and Biochemical characteristics of isolated strains

| **Characteristics** | **Results** |
| --- | --- |
| Siderophore (%) | 89.20±0.65 |
| Indole acetic acid (µg ml^-1^) | 48.56 |
| NH_3_ Production | +ve |
| HCN Production | +ve |
| Phosphate Solubilization (mm) | 7.8 |
| Gram staining and Shape | Positive and Rod Shape |
| Motility | Motile |
| Catalase test | Positive |
| Citrate test | Positive |

Data is a mean of Triplicate± Standard deviation
